# *FOXD1* mutations are related to repeated implantation failure, intra-uterine growth restriction and preeclampsia

**DOI:** 10.1186/s10020-019-0104-3

**Published:** 2019-08-08

**Authors:** Paula Quintero-Ronderos, Karen Marcela Jiménez, Clara Esteban-Pérez, Diego A. Ojeda, Sandra Bello, Dora Janeth Fonseca, María Alejandra Coronel, Harold Moreno-Ortiz, Diana Carolina Sierra-Díaz, Elkin Lucena, Sandrine Barbaux, Daniel Vaiman, Paul Laissue

**Affiliations:** 1 Center For Research in Genetics and Genomics-CIGGUR. GENIUROS Research Group. School of Medicine and Health Sciences, Universidad del Rosario, Bogotá, Colombia; 2 Fertility and Sterility Colombian Center, Department of Reproductive Genetics, Bogotá, Colombia; 3 Clinical Neurosciences and Psychiatry, Clinical and Experimental Sciences, Faculty of Medicine, University of Southampton, Southampton, UK., Southampton, United Kingdom; 4 Inserm U1016, CNRS UMR8104, Institut Cochin, équipe FGTB, 24, rue du faubourg Saint-Jacques, 75014 Paris, France

## Abstract

**Background:**

Human reproductive disorders consist of frequently occurring dysfunctions including a broad range of phenotypes affecting fertility and women’s health during pregnancy. Several female-related diseases have been associated with hypofertility/infertility phenotypes, such as recurrent pregnancy loss (RPL). Other occurring diseases may be life-threatening for the mother and foetus, such as preeclampsia (PE) and intra-uterine growth restriction (IUGR). FOXD1 was defined as a major molecule involved in embryo implantation in mice and humans by regulating endometrial/placental genes. *FOXD1* mutations in human species have been functionally linked to RPL’s origin.

**Methods:**

*FOXD1* gene mutation screening, in 158 patients affected by PE, IUGR, RPL and repeated implantation failure (RIF), by direct sequencing and bioinformatics analysis. Plasmid constructs including *FOXD1* mutations were used to perform in vitro gene reporter assays.

**Results:**

Nine non-synonymous sequence variants were identified. Functional experiments revealed that p.His267Tyr and p.Arg57del led to disturbances of promoter transcriptional activity (*C3* and *PlGF* genes). The FOXD1 p.Ala356Gly and p.Ile364Met deleterious mutations (previously found in RPL patients) have been identified in the present work in women suffering PE and IUGR.

**Conclusions:**

Our results argue in favour of FOXD1 mutations’ central role in RPL, RIF, IUGR and PE pathogenesis via *C3* and *PlGF* regulation and they describe, for the first time, a functional link between *FOXD1* and implantation/placental diseases. *FOXD1* could therefore be used in clinical environments as a molecular biomarker for these diseases in the near future.

**Keywords:**

Recurrent pregnancy loss, Preeclampsia, Intra-uterine growth restriction, FOXD1

**Electronic supplementary material:**

The online version of this article (10.1186/s10020-019-0104-3) contains supplementary material, which is available to authorized users.

## Background

Human reproductive disorders consist of frequently occurring dysfunctions including a broad range of phenotypes affecting fertility and women’s health during pregnancy. Several female-related diseases have been associated with hypofertility/infertility phenotypes, most of which can affect the ovaries (e.g. primary ovarian insufficiency-POI), the hormonal system (e.g. polycystic ovary syndrome-PCOS), the fallopian tubes (e.g. obstruction) and/or the endometrium (e.g. recurrent pregnancy loss-RPL- and endometriosis) (Laissue, 2018; Smith et al., 2003). Other commonly occurring diseases may be life-threatening for the mother and foetus, such as preeclampsia (PE) and intra-uterine growth restriction (IUGR) both of which causes important physiological changes during pregnancy.

RPL (which affects 2–5% of all pregnancies) has been clinically defined as being at least three pregnancy losses occurring before the 20th week of gestation (El Hachem et al., 2017). Its aetiology is still poorly understood as, although several causes have been described, > 50% of cases are considered idiopathic; such scenario pinpoints the potential participation of a genetic component related to its origin. Various tools have been used for identifying loci and sequence variants related to this disease’s aetiology, such as genome-wide association studies (GWAS), Sanger and next generation sequencing (NGS), linkage analysis and DNA methylation status assessment (Kolte et al., 2011; Li Wang et al., 2010; Pereza et al., 2017; Vaiman, 2015). However, the definitive association of genetic variants or epigenetic modifications with the phenotype has rarely been validated by functional tests.

PE is another frequently occurring disease (~ 5% of pregnancies) which is clinically characterised by pregnancy-induced hypertension and proteinuria, making it one of the main causes of pregnancy-related maternal and foetal morbimortality. Although various pathophysiological mechanisms have been described, PE’s precise aetiology remains unknown (Chaiworapongsa et al., 2014). Identifying early diagnostic/prognostic biomarkers has become a relevant focus for research as PE’s clinical signs and symptoms appear during the third trimester of gestation. More than 15 loci have been mapped and positional cloning has led to interesting PE candidates being identified, such as *ACVR2A*, *TNFSF13B, EPAS1* and *STOX1* (Chelbi et al., 2013; Jebbink et al., 2012) (and references therein). STOX1, a transcription factor, has been defined as a key regulator of placental genes and its mutations have been related to PE pathogenesis (van Dijk et al., 2010; Vaiman and Miralles, 2016). Interestingly, *Stox1* overexpression in mice has led to placental and endothelial cell dysfunction, PE, IUGR and cardiovascular injury (Collinot et al., 2018; Ducat et al., 2016). Some sequence variants located on additional genes (e.g. *SERPINA8*, *MMP9*, *VEGF* and *TNFα*) have been found to increase the risk of PE (Chelbi et al., 2013). Regarding IUGR, maternal placental and foetal genes have been proposed as relevant pathophysiology actors (*SERPINA3*, *PlGF*, *BCL2*, *BAX*, *IGF1/IGF2*, *VEGF*, *STOX1*, *FV*, *SVCAM1* and *ADMA*) (Sharma et al., 2017).

Interestingly, the participation of common genes and molecular pathways in IUGR, PE and RPL pathophysiology argues in favour of the potential existence of central regulatory actors (e.g. transcription factors) involved in these disorders’ aetiopathology.

A series of studies using a genetic mouse model of interspecific congenic strains allowed us to map quantitative trait loci (QTL) related to embryo resorption (a phenotype analogous to RPL in humans) to short chromosome regions (Laissue et al., 2016, 2009; Vatin et al., 2012). One of these regions was found to contain *FOXD1*, encoding a forkhead transcription factor, shown to be involved in the regulation of embryo implantation in mice (Laissue et al., 2016, 2009; Quintero-Ronderos and Laissue, 2018). The mouse Foxd1-Thr152Ala variant (carried naturally by the *Musspretus* species), when expressed in the C57BL/6 J genetic background, was associated to embryo resorption and massive deregulation of the expression of placental and endometrial genes (Laissue et al., 2016). *FOXD1* mutations in humans have now been functionally linked to RPL’s origin, thereby constituting a diagnostically useful molecular biomarker (Laissue et al., 2016; Quintero-Ronderos and Laissue, 2018).

Herein, we describe novel *FOXD1* gene mutations identified through the screening of 158 patients affected by PE, IUGR, RPL and repeated implantation failure (RIF) following in vitro fertilisation. Nine non-synonymous sequence variants were identified, two of which (p.His267Tyr found in one RIF patient and p.Arg57del in one IUGR woman) represented novel and coherent candidates for in vitro testing. Functional experiments revealed that both led to an increased *C3* (complement C3) promoter transcriptional activity. In addition, we found increased FOXD1-p.Arg57del variant transactivation capacity on the *PlGF* (placental growth factor) promoter. The FOXD1 p.Ala356Gly and p.Ile364Met mutations (previously found in RPL patients) have also been identified in the present work in women with PE and IUGR and with isolated IUGR, respectively.

Our results provide new evidence of *FOXD1*’s central role in endometrium and placental physiology as we show, for the first time, that besides its involvement in RPL its mutations contribute to RIF, IUGR and PE. *FOXD1* could therefore be used in clinical environments as a molecular biomarker for these diseases in the near future.

## Methods

### Patients and controls

The study population consisted of 158 women suffering from different reproductive disorders: RPL (*n* = 31), RIF (*n* = 30), IUGR (*n* = 39), PE (n = 31), PE and IUGR (PE/IUGR) (*n* = 27). The RPL and RIF patients were from Colombian origin, whilst those suffering IUGR, PE or PE/IUGR were French (Table 1). The control groups consisted of 203 Colombian and 361 French women lacking a clinical history of reproductive disorders (also see below).

**Table 1:** *FOXD1* ORF sequencing in RPL, RIF, IUGR and PE patients

Colombian RPL patients								
DNA	Protein	Rs	Patients	Controls*	MAF (gnomAD)***	SIFT	PolyPhen	Evolutive conservation
c.263C > G	p.Ala88Gly	rs7705335	5/31	11/203	0.1080	Tolerate (0,52)	Benign (0)	Yes
c.308G > A	p.Gly103Asp	rs1369199745	1/31	2/203	0.0000	Tolerate (0,46)	Benign (0,334)	Yes
Colombian RIF patients								
DNA	Protein	Rs	Patients	Controls*	MAF (gnomAD)***	SIFT	PolyPhen	Evolutive conservation
c.263C > G	p.Ala88Gly	rs7705335	2/30	11/203	0,1080	Tolerate (0,99)	Benign (0)	Yes
c.308G > A	p.Gly103Asp	rs1369199745	1/30	2/203	0.0000	Tolerate (0,46)	Benign (0,334)	Yes
**c.799C > T**	**p.His267Tyr**	**___**	**1/30**	**0/203**	**__**	**Deleterious (0,04)**	**Possibly deleterious (0,69)**	**Yes**
c.976G > A	p.Ala326Thr	rs552595262	1/30	5/203	0.0000	Deleterious (0,07)	Possibly deleterious (0,69)	Yes
French IUGR patients								
DNA	Protein	Rs	Patients	Controls**	MAF (gnomAD)***	SIFT	PolyPhen	Evolutive conservation
**c.168_170delGCG**	**p.Arg57del**	**rs750578392**	**1/39**	**0/361**	**0.0001**	**__**	**__**	**Yes**
c.263C > G	p.Ala88Gly	rs7705335	4/39	31/361	0.1080	Tolerate (0,99)	Benign (0)	Yes
c.976G > A	p.Ala326Thr	rs552595262	1/39	11/361	0.0000	Deleterious (0,07)	Possibly deleterious (0,69)	Yes
c.1092C > G	p.Ile364Met	rs992724147	1/39	0/361	0.0000	Deleterious (0)	Deleterious (0,99)	Yes
c.1146_1158delCAGGCCGCCGCC	p.Gln383_Ala387delQAAA	rs771204220	4/39	18/361	0.0308	__	__	NA
French PE patients								
DNA	Protein	Rs	Patients	Controls**	MAF (gnomAD)***	SIFT	PolyPhen	Evolutive conservation
c.263C > G	p.Ala88Gly	rs7705335	6/31	31/361	0.1080	Tolerate (0,99)	Benign (0)	Yes
c.976G > A	p.Ala326Thr	rs552595262	2/31	11/361	0.0000	Deleterious (0,07)	Possibly deleterious (0,69)	Yes
French PE + IUGR patients								
DNA	Protein	Rs	Patients	Controls**	MAF (gnomAD)***	SIFT	PolyPhen	Evolutive conservation
c.263C > G	p.Ala88Gly	rs7705335	1/27	31/361	0.1080	Tolerate (0,99)	Benign (0)	Yes
c.1067C > G	p.Ala356Gly	rs917127030	1/27	0/361	0.0000	Deleterious (0,01)	Benign (0,001)	Yes
c.1187C > T	p.Pro396Leu	rs540644822	1/27	2/361	0,0042	Tolerate (0,2)	Benign (0,1)	Yes

*Colombian controls, **French controls, ***gnomAD v2.1.1 (controls)

Bold data are significant

The unrelated Colombian RPL patients (*n* = 31) attended the Centre for Research in Genetics and Genomics (CIGGUR-Universidad del Rosario, Bogotá, Colombia). They had suffered 3 or more consecutive pregnancy losses and had normal 46, XX karyotypes. They had no clinical history of coagulation dysfunction, uterine anomalies, autoimmunity (e.g. antiphospholipid syndrome), infection, endocrine and/or metabolic disorders (excluded by biochemical tests). All cases had no background of consanguinity or reproductive diseases.

The Colombian RIF patients (*n* = 30) were attending the Colombian Fertility and Sterility Centre (Cecolfes, Bogotá, Colombia). The inclusion criteria referred to women having suffered two or more RIF after at least 2 consecutive cycle of IVF or ICSI in which a high-quality embryo had been transferred during each cycle (Rinehart, 2007). β-HCG serum levels were followed-up for monitoring implantation success. Maternal > 40 year-old patients suffering uterine anomalies, miomatosis, hydrosalpinx, having an abnormal karyotype, male-related abnormality factors (e.g. oligospermia, azoospermia), suffering endocrine and coagulation and autoimmunity diseases were excluded from the study.

The French IUGR (*n* = 39), PE (*n* = 31) and PE and IUGR (PE/IUGR (*n* = 27)) patients were attending The Institut Cochin (Paris, France). Inclusion criteria for PE were systolic pressure above 140 mmHg, diastolic pressure above 90 mmHg and proteinuria above 0.3 g per day. The inclusion criteria used for IUGR were reduction of fetal growth during gestation with a birth weight below the 10th percentile according to Lubchenco growth curves. The exclusion criteria included diabetes, chromosomal and fetal malformations, maternal infections, aspirin treatment. The control group for Colombian patients consisted of 203 women (from the same ethnical origin) over 50 years-old having had at least one live birth child without antecedents of medical complications during pregnancy and lacking hypertensive disorders. Regarding the French controls, we used data previously reported by our group (Laissue et al., 2016). In that study, *FOXD1* was sequenced in 271 French controls lacking antecedents of obstetrical disorders. In the present study we have increased the amount of French controls to 361 using the same DNA bank. Blood samples were collected from all patients and controls using standard procedures.

All participating individuals signed an informed consent form. All this study’s experimental steps were approved by the Universidad del Rosario’s and Institut Cochin’s Ethics Committees and the study was conducted in line with the Declaration of Helsinki.

### *FOXD1* sequencing and bioinformatics analysis

DNA was extracted from all patients and controls’ whole blood samples using the salting-out method. *FOXD1* amplification and sequencing has been described previously (Laissue et al., 2016). Amplicons were purified using shrimp alkaline phosphatase and exonuclease I. Internal primers were used for sequencing. The sequences were compared to that of the FOXD1 wild type version (ENSG00000251493). Primer sequences, PCR and sequencing technical conditions have been included as supplementary information (Additional file 1). The novel variants were screened in the gnomAD database (https://gnomad.broadinstitute.org). We also compared the variants’ allele frequencies identified in patients to those from their ethnically-matched controls (Colombian population). SIFT and PolyPhen-2 bioinformatics tools were used for assessing the novel FOXD1-p.His267Tyr missense variant’s potentially harmful effects. FOXD1 proteins from orthologous species (*Monodelphis domestica*, *Pan troglodytes*, *Sus scrofa*, *Cebus capucinus imitator*, *Odobenus rosmarus divergens*, *Delphinapterus leucas*) were aligned to determine potential conservation of His ^267^ during evolution.

### Plasmid constructs and in vitro gene reporter assays

The complete *FOXD1* ORF WT and mutant versions (p.His267Tyr and p.Arg57del) were inserted into the pcDNA 3.1 Zeo (+) vector (Invitrogen, Carlsbad, CA, USA). The *C3* promoter (− 792 to − 63 bp upstream of the initial ATG start codon) was inserted into pGL4.22[luc2CP/Puro] (Invitrogen, Carlsbad, CA, USA). A digestion-ligation protocol was used for *FOXD1* and *C3* promoter cloning, using 5′-KpnI and XhoI-3′ endonucleases and T4 DNA ligase (Invitrogen). The construct containing the *PlGF* promoter region was previously described (Laissue et al., 2016). All constructs were sequenced to exclude potentially unexpected PCR-induced mutations.

COS-7 cells were cultured in Dulbecco’s modified Eagle medium/Ham’s Nutrient Mixture F12 (DMEM/F12, Gibco) containing 10% foetal bovine serum (FBS-Biowest) and 1% penicillin/streptomycin (Invitrogen-Gibco, Carlsbad, CA, U.S.A) at 37 °C in a 5% CO2 atmosphere. Cells were seeded at 50,000 cells/well in 24-well culture dishes and incubated at 37 °C in 5% CO2 for 24 h. Cells were co-transfected using Fugene (Promega, Madison, WI, USA) reagent in a serum-free medium with 800 ng of constructs including the *C3* or *PlGF* promoters, 500 ng FOXD1-WT or mutant versions (c.168_170delGCG; p.Arg57del or c.799C > T-p.His267Tyr) and 30 ng Renilla for 48 h. Negative control involved co-transfection with pcDNA 3.1 Zeo (+) empty vector.

*C3* and *PlGF* transcriptional activities, in response to the WT or mutant versions of FOXD1, were measured 48 h after transfection using the Dual-Luciferase Reporter Assay System, following the manufacturer’s instructions (Promega, Madison, WI, USA). The luciferase activity reported for each experiment was divided by Renilla activity to obtain RLUs values. Each experiment was repeated three times in sixplicate. Student’s t-test was used for estimating the statistical significance between WT and mutant conditions.

## Results

### *FOXD1* genotyping and bioinformatics analysis

Sequence analysis revealed 9 heterozygous non-synonymous sequence variants (Table 1 gives *FOXD1* genotyping results). Among those, four, c.168_170delGCG (p.Arg57del), c.799C > T (p.His267Tyr), c.1067C > G (p.Ala356Gly) and c.1092C > G (p.Ile364Met) were rare as they displayed a very low minor allele frequency (MAF) in public databases of SNPs (e.g. gnomAD). In addition, they were absent in the control group described by Laissue et al., (2016) nor in the present work’s control groups. The c.168_170delGCG (p.Arg57del) and c.799C > T (p.His267Tyr) variants had not been described previously. The p.His267Tyr variant was found in a Colombian RIF patient whilst the p.Arg57del variant was carried by a French IUGR patient. The c.1067C > G (p.Ala356Gly) and c.1092C > G (p.Ile364Met) variants have been previously reported in RPL women (Laissue et al., 2016). Here, one French IUGR/PE patient had the p.Ala356Gly mutation while one IUGR woman carried the p.Ile364Met mutation. The remaining variants were considered to be polymorphisms, having > 1% MAF in the gnomAD SNP database and/or were present in control populations (i.e. 361 French or 203 Colombian women from the present research) (Laissue et al., 2016). SIFT and PolyPhen prediction tools gave scores compatible with a harmful effect for the p.His267Tyr variant (Table 1). This variant’s protein sequence alignment suggested strict His^267^ residue conservation during the species’ evolution (Additional file 2: Figure S1).

### Luciferase gene reporter assays

**Fig. 1:**
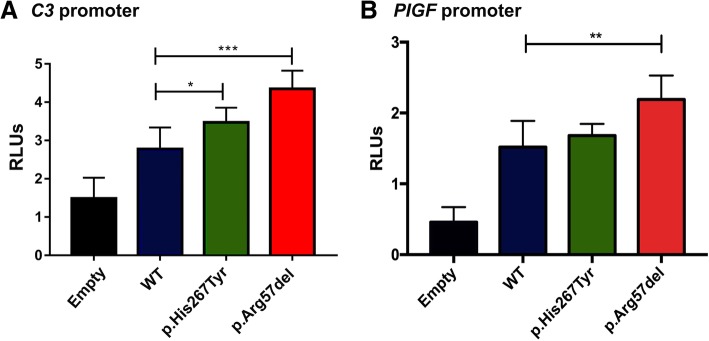
Transactivation properties of FOXD1-WT and mutant versions on *C3* and *PlGF* promoters. The FOXD1-WT version overexpression allowed to transactivate the *C3* and *PlGF* promoters in luciferase gene reporter assays (C3: WT vs empty vector, 1.9-fold, *p* = 0.0024; PlGF: WT vs empty vector, 3-fold, *p* = 1.3 × 10^− 5^) (**a** and **b** panels). **a** Compared to that of the WT version, the FOXD1 p.His267Tyr and p.Arg57del mutations increased significantly *C3* transcriptional activity (1.25-fold, *p* = 0.03 and 1.5-fold, *p* = 0.0004, respectively). **b** The FOXD1-p.Arg57del mutation increased PlGF transcriptional activity (1.4 fold, *p =* 0.002) compared to that for the FOXD1-WT counterpart. RLU: relative luciferase units. (*): *p* < 0.05; (***): *p* < 0.001

The FOXD1-WT version overexpression allowed to transactivate the *C3* and *PlGF* promoters in luciferase gene reporter assays (C3: WT vs empty vector, 1.9-fold, *p* = 0.0024; PlGF: WT vs empty vector, 3-fold, *p* = 1.3 × 10^− 5^), as reported before by Laissue et al. (Laissue et al. 2016) (Fig. 1). Compared to that of the WT version, the FOXD1 p.His267Tyr and p.Arg57del mutations increased significantly *C3* transcriptional activity (1.25-fold, *p* = 0.03 and 1.5-fold, *p* = 0.0004, respectively). The FOXD1-p.Arg57del mutation increased PlGF transcriptional activity (1.4 fold, *p =* 0.002) compared to that for the FOXD1-WT counterpart.

## Discussion

IUGR and PE are two complex diseases which are frequently associated with maternal and foetal complications during pregnancy. A clear association between these disorders has been documented, as women suffering PE have an increased risk (up to 4-fold) of being affected by IUGR (Fox et al., 2014; Srinivas et al., 2009). Conversely, IUGR-affected individuals have an increased risk of being affected by PE (Mitani et al., 2009). PE and IUGR share pathophysiological mechanisms affecting the placenta and endometrial tissues, such as hypoxia, thrombosis, ischemia, impaired angiogenesis and inflammation (Armaly et al., 2018; Collinot et al., 2018; Garrido-Gomez et al., 2017; Gurugubelli and Vishnu, 2018; Shamshirsaz et al., 2012; Sharma et al., 2017). Several molecular pathways thus become simultaneously dysregulated, which may partly result from the dysfunction of key transcription factors acting in the endometrium and the placenta. Particular interest in FOXD1 has been highlighted as it has been shown to play a central role in mammalian embryo implantation and pregnancy maintenance (Laissue et al., 2016, 2009). FOXD1 mutations have led to embryo resorption in mice and RPL in humans by perturbing transcriptional networks in the endometrium and placenta. It was thus considered that *FOXD1* was a coherent candidate gene in the present study as it is potentially related to other female reproductive phenotypes, such as RIF, IUGR and PE.

We focused our attention on FOXD1-p.His267Tyr and p.Arg57del from the 9 non-synonymous sequence variants identified in the present study since they are rare and had not been described previously in RPL women (Laissue et al., 2016) (Table 1). The c.799C > T (p.His267Tyr) variant was carried by a Colombian RIF patient. Since the Colombian population consists of a particular ethnic admixture, and its genetic composition/variability is not widely represented in public SNP databases, we screened this variant in a panel of 203 ethnically-matched controls. The variant was not found in this control population, thereby arguing in favour of an association with the disease’s aetiology. Furthermore, the His^267^ residue has been conserved during mammalian species’ evolution, strongly suggesting functional relevance (Additional file 1). Accordingly, SIFT and PolyPhen bioinformatics’ prediction tools gave scores compatible with a harmful effect (Table 1). Furthermore, replacing a histidine (His) with a tyrosine (Tyr) has been predicted to be potentially deleterious, since as His is an amino acid which is electrically charged with basic side chains, whilst Tyr is a large aromatic polar uncharged molecule. The p.His267Tyr mutation could thus have led to local or global changes regarding FOXD1’s physiochemical properties, thereby contributing to transcriptional disturbances.

We used a gene reporter system to explore this hypothesis as it facilitated assessing FOXD1’s transactivation capability regarding the *C3* and PlGF promoters. C3 belongs to the complement system family of proteins which has at least 50 members and can be activated in several tissues by different mechanisms (Regal et al., 2015). Interestingly, complement factors (including C3) act at the crossroads of endometrium/placenta development and physiology, meaning that they can be considered key molecules potentially involved in various female reproductive disorders (Laissue et al., 2016; Regal et al., 2017, 2015). Recurrent studies in animal models hint to a central effect of C3 deregulation in placental pathophysiology (Girardi, 2018; Girardi et al., 2015; Qing et al., 2011; Wang et al., 2012).

In vitro, we showed that the protein’s WT version was able to transactivate the *C3* promoter (1.9-fold, *p* = 0.024) (Fig. 1). The FOXD1-p.His267T and the pArg57del mutations led to statistically significant increases in *C3* transcription activity compared to that induced by the WT version. This finding reinforced those described previously for the FOXD1-p.Ile364Met and p.429AlaAla mutations identified in RPL women, arguing in favour of this variant’s functional contribution to the phenotype (Laissue et al., 2016).

High *C3* levels have been recorded in women having suffered three pregnancy losses, which might be linked to other inflammatory-related molecules’ local (endometrium and placental tissues) expressional disturbances. Interestingly, increased complement activation has been recorded in human placentas following spontaneous abortion, while CD46 and CD55 (complement regulators) became reduced (Banadakoppa et al., 2014; Regal et al., 2015). Here, we have identified the FOXD1-p.His267Tyr mutation in a RIF patient consistently with the hypothesis that *FOXD1* plays an essential role in early pregnancy maintenance. This is consistent with our previous observations where the 66H-IRCS strain of mice (which carries the *M. spretus*–derived Foxd1-Thr152Ala mutation) presents with high rates of early embryonic death (Laissue et al., 2009).

Regarding the FOXD1-p.Arg57del mutation (which we identified in an IUGR patient), we also considered it as potentially having a functional impact because it had a low MAF in the gnomAD database. Similarly to other harmful FOXD1 missense mutations, FOXD1-p.Arg57del may lead to the protein’s three-dimensional conformational changes and functional disturbances. The FOXD1-p.Arg57del mutation increased 1.5-fold *C3* promoter transcriptional activity. Although C3 levels have not been widely studied in women affected by IUGR, due to its relevant role during placental physiology we consider that the transcriptional increase observed in our experiments might also be found in vivo.

FOXD1 has already been shown to be a regulator of *PlGF* in mice and humans (Zhang et al., 2003, Laissue et al., 2016). We observed increased *PlGF* transcriptional activity (1.4 fold, *p* = 0.002) with the FOXD1-p.Arg57del mutation compared to that for the FOXD1-WT counterpart, thereby arguing in favour of a potential placental dysfunction leading to IUGR. Low plasmatic PlGF levels have been reported in PE women and it has been seen that FOXD1 mutations have led to reduced induction capacity on the PlGF promoter in recurrent pregnancy loss patients, whilst *PlGF* overexpression has been linked to enhanced angiogenesis in tumours (Laissue et al., 2016, Chau et al., 2017 and references therein). These findings and the results of the present work, suggest that fine-tuning *PlGF* expression is an essential condition contributing to placental/endometrial physiology; indeed its transcriptional dysregulation may contribute to different diseases pathogenesis.

Interestingly, two FOXD1 previously identified mutations (p.Ile364Met, p.Ala356Gly) were re-identified in the present study in IUGR patients. We have previously found them in RPL women and shown that they led to *C3* promoter transactivation disturbances (Laissue et al., 2016). Indeed, similarly to that observed in our present FOXD1-p.His267Tyr and p.Arg57del experiments, the FOXD1-p.Ile364Met mutation also increased *C3* promoter transcription activity ~ 5 fold (Laissue et al., 2016). These findings argue in favour of FOXD1 mutations possibly contributing to IUGR pathogenesis.

Surprisingly, contrary to that observed for the FOXD1-p.Arg57del mutation, FOXD1-p.Ala356Gly has been reported to decrease *C3* promoter transcription activity (Laissue et al., 2016). Although complement cascade activation has been observed in PE patients, it has been postulated that the fine tuning of *C3* expression may be an important factor regarding physiological gestation in mice and humans (Chow et al., 2009; Laissue et al., 2016; Lynch et al., 2012, 2011, 2008; Regal et al., 2017). *C3* expression disturbances (up or down-regulation) due to FOXD1 mutations over/under specific thresholds might therefore contribute to RPL, PE, and/or IUGR. The functional differences amongst FOXD1 mutations might be related to specific physicochemical modifications triggered by particular amino acid changes and/or secondary to regulatory networks’ inherent downstream complexity. It should be also taken into account that other genetic (e.g. variants in other genes) and epigenetic (e.g. imprinting of paternal alleles, or consequences to variable environmental exposures) changes may modify FOXD1 mutations’ phenotypic effect.

Taken together, our results argue in favour of FOXD1 mutations’ central role in RPL, RIF, IUGR and PE pathogenesis via *C3* regulation (Laissue et al., 2016). We consider that *FOXD1* should be genotyped in larger panels of patients to establish an accurate genotype-phenotype correlation and to justify proposing it as a reliable, clinically useful biomarker.

## Conclusions

Taken together, our results argue in favour of FOXD1 mutations’ central role in RPL, RIF, IUGR and PE pathogenesis via *C3* regulation [18]. Although several functionally harmful *FOXD1* mutations have been described, a well-documented genotype-phenotype correlation remains to be discovered. This should help clinicians in making more accurate diagnosis/predicting several pregnancy-related disorders. Identifying new mutations and their functional impact may lead to using *FOXD1* in the near future as a clinically useful biomarker.

## Additional files

Additional file 1: **Supplementary Methods.** DNA extraction, *FOXD1* amplification and direct sequencing. (DOCX 19 kb)

Additional file 2: **Figure S1.** Interspecific alignment of FOXD1 in vertebrate species. The His^267^ residue is highlighted in pink. (PDF 417 kb)

## Data Availability

The data analysed during the current study are available from the corresponding author on reasonable request.

## Abbreviations

GWAS: Genome-wide association studies
His: Histidine
IUGR: Intra-uterine growth restriction
MAF: Minor allele frequency
NGS: Next generation sequencing
PCOS: Polycystic ovary syndrome
PE: Preeclampsia
POI: Primary ovarian insufficiency
RIF: Repeated implantation failure
RPL: Recurrent pregnancy loss
SNP: Single nucleotide polymorphism
Tyr: Tyrosine
WT: Wild-type
