# Supplementary material for: FOXD1 mutations are related to repeated implantation failure, intra-uterine growth restriction and preeclampsia
Source: Mol Med. 2019 Aug 8;25:37. doi: 10.1186/s10020-019-0104-3 (PMC6688323; doi:10.1186/s10020-019-0104-3)
Supplement: Supplementary file 1 — Supplementary Methods. DNA extraction, FOXD1 amplification and direct sequencing. (DOCX 19 kb) [file 10020_2019_104_MOESM1_ESM.docx]

**Additional file 1**

**Supplementary methods**

**DNA extraction**

The salting-out method was used for extracting DNA from all patients and controls’ whole blood samples. The quality of the DNA was evaluated through measurement of absorbance (OD260/OD280 and OD260/OD230) with a NanoDrop^TM^ One/One^C^ (ThermoFisher Scientific, Waltham, MA, USA).

***FOXD1* amplification**

The sequence (monoexonic gene) of the transcription factor *FOXD1* is composed by 1398nt and within its sequence there are GC rich regions. In the position 736 bp from the start codon (bold), there is a G-repetition that allow the formation of a hairpin structure. For these reasons, the FOXD1 amplification is challenging and should be performed with DMSO 5% and in two fragments. A good DNA quality is required

The *FOXD1* sequencing is performed with a Forward and Reverse primer for each fragment (see below)

***FOXD1* Nucleotide Sequence (1398 nt):** **NM_004472.2**

cggagcccagggagcgcagcatccgcgcggctaccgcggcggcgcaggagttataaagtcggcgcgcgagactccgccgccacccggcagccccggcgcagctccggcagccgcagtcgcagcgcccccagcgtggcgccccccggccgggcctgccgcccgggacccgggctggggcgcagagggagcccggagcccggcgcccccatgcgccgccccgccgccgccgcgccacagct

ATGACCCTGAGCACTGAGATGTCCGATGCCTCTGGCCTCGCCGAGGAAACAGACATCGACGTGGTGGGGGAGGGCGAGGACGAAGAAGACGAGGAAGAGGAGGACGACGACGAGGGCGGCGGTGGCGGGCCCCGGCTGGCTGTCCCCGCGCAGCGGCGGCGGCGGCGGCGCTCGTACGCCGGGGAGGACGAGCTGGAGGATCTGGAGGAGGAGGAGGACGACGATGACATCCTGCTGGCCCCGCCTGCTGGGGGCTCCCCGGCGCCCCCGGGCCCGGCCCCGGCGGCGGGGGCAGGAGCCGGTGGGGGCGGCGGCGGCGGCGGCGCGGGCGGCGGCGGGAGCGCGGGTAGCGGCGCCAAGAACCCGCTGGTGAAGCCGCCCTACTCGTATATCGCGCTCATCACTATGGCCATCCTGCAGAGCCCCAAGAAGCGGCTGACGCTGAGCGAGATCTGTGAGTTCATCAGCGGCCGCTTCCCCTACTACCGGGAGAAGTTCCCCGCCTGGCAGAACAGCATCCGCCACAACCTCTCGCTCAACGACTGCTTCGTCAAGATCCCCCGCGAGCCCGGCAACCCGGGCAAGGGCAACTACTGGACGCTGGACCCGGAGTCCGCCGACATGTTCGACAACGGCAGCTTCCTGCGCCGGAGGAAGCGCTTCAAGCGGCAGCCGCTGCTCCCACCCAACGCCGCGGCCGCCGAGTCTCTGCTGCTGCGCGGCGCGGGAGCCGCA**GGGGG**CGCGGGCGACCCGGCAGCCGCCGCCGCGCTCTTCCCGCCCGCGCCCCCGCCGCCCCCGCATGCCTACGGCTACGGCCCCTACGGCTGCGGCTACGGCCTGCAGCTGCCGCCTTACGCGCCGCCCTCGGCCCTCTTCGCCGCCGCAGCGGCCGCCGCCGCCGCCGCCGCCTTCCACCCGCACTCGCCCCCGCCGCCCCCGCCACCGCACGGCGCGGCCGCCGAGCTGGCCCGGACCGCCTTCGGCTACCGGCCGCACCCGCTCGGCGCCGCCCTACCCGGCCCCCTGCCGGCCTCCGCGGCCAAGGCGGGCGGCCCGGGCGCCTCAGCGCTGGCGCGCTCGCCCTTCTCCATCGAGAGCATCATCGGGGGCAGCTTGGGCCCGGCCGCCGCTGCCGCCGCCGCCGCGCAGGCCGCCGCCGCCGCTCAGGCCTCGCCCTCGCCCTCGCCGGTGGCGGCGCCGCCAGCTCCCGGATCCAGCGGAGGAGGCTGCGCGGCGCAGGCGGCCGTGGGCCCGGCGGCCGCGCTCACCCGATCCCTCGTGGCCGCCGCGGCCGCCGCCGCCTCCTCAGTCTCCTCGTCCGCCGCCTTGGGGACTCTGCACCAAGGGACTGCCCTGTCCAGTGTCGAGAACTTTACTGCTAGGATTTCCAATTGTTAA

taacgctatgttagcgcgctcgaggaagaaggtaggaatcccggctccttttctcgtcttggtggttcggtgttttgttcgctcctccaggcgcggcccctctcgacctcgcgcgcccattttcgccgctgcgaattctcggacaaaactgtcaacagcccgggcgcgcctttt

CGCA**GGGGG**CGCGGGC 🡪 Hairpin

- **Primers Fragment 1 (924 bp)**
  - HuFOXD1-1Fb: cggcgcaggagttataaagtcggcg
  - HuFOXD1-1Rb: CGCCGCGCAGCAGCAGAGACTCG
- **Primers Fragment 2 (974 bp)**
  - HuFOXD1-2Fb: GCAACTACTGGACGCTGGACCCGGAG
  - HuFOXD1-2Rb: cgggctgttgacagttttgtccgag

**Mix for a 25 µl reaction volume**

| **Components** | **Volume** | **Final Concentration** |
| --- | --- | --- |
| PCR Master Mix 2X | 12.5 µl | 1X |
| Primer Forward 10 µM | 1.7 µl | 0.7 µM |
| Primer Reverse 10 µM | 1.7 µl | 0.7 µM |
| DNA template | --- | 100 ng |
| DMSO | 1.25 µl | 5 % |
| Nuclease-Free Water to | 25 µl | --- |

**PCR amplification protocol (Touchdown PCR)**

- **Fragment 1**

| **Step** | **Temperature** | **Time** |
| --- | --- | --- |
| **1 cycle** | | |
| Initial denaturation | 95 °C | 12 min |
| **14 cycles** | | |
| Denaturation | 95 °C | 40 seg |
| Annealing | 67 °C | 30 seg |
| Extension | 72 °C | 50 seg |
| **12 cycles** | | |
| Denaturation | 95 °C | 40 seg |
| Annealing | 66 °C | 30 seg |
| Extension | 72 °C | 50 seg |
| **9 cycles** | | |
| Denaturation | 95 °C | 40 seg |
| Annealing | 65 °C | 30 seg |
| Extension | 72 °C | 50 seg |
| **1 cycle** | | |
| Final extension | 72 °C | 10 min |

- **Fragment 2**

| **Step** | **Temperature** | **Time** |
| --- | --- | --- |
| **1 cycle** | | |
| Initial denaturation | 95 °C | 12 min |
| **4 cycles** | | |
| Denaturation | 95 °C | 40 seg |
| Annealing | 69 °C | 30 seg |
| Extension | 72 °C | 50 seg |
| **4 cycles** | | |
| Denaturation | 95 °C | 40 seg |
| Annealing | 68 °C | 30 seg |
| Extension | 72 °C | 50 seg |
| **13 cycles** | | |
| Denaturation | 95 °C | 40 seg |
| Annealing | 67 °C | 30 seg |
| Extension | 72 °C | 50 seg |
| **14 cycles** | | |
| Denaturation | 95 °C | 40 seg |
| Annealing | 66 °C | 30 seg |
| Extension | 72 °C | 50 seg |
| **1 cycle** | | |
| Final extension | 72 °C | 10 min |

**FOXD1 Sequencing protocol**

Our amplification products were sent to ELIM Biopharma, Inc for sequencing.

1. Each PCR product should be sent with a final concentration of 600 ng in a volume of 10 µl.
2. The primers used for sequencing are:
   - Fragment 1: HuFOXD1-1Fb: cggcgcaggagttataaagtcggcg

HuFOXD1-1Rb: CGCCGCGCAGCAGCAGAGACTCG

- - Fragment 2: HuFOXD1-2Fb: GCAACTACTGGACGCTGGACCCGGAG

HuFOXD1-2Rb: cgggctgttgacagttttgtccgag

1. Each primer should be sent with a final concentration of 3 µM and a minimum of 3µl of each primer is required for every PCR product.
