# Supplementary material for: FOXD1 mutations are related to repeated implantation failure, intra-uterine growth restriction and preeclampsia
Source: Mol Med. 2019 Aug 8;25:37. doi: 10.1186/s10020-019-0104-3 (PMC6688323; doi:10.1186/s10020-019-0104-3)
Supplement: Supplementary file 2 — Figure S1. Interspecific alignment of FOXD1 in vertebrate species. The His267 residue is highlighted in pink. (PDF 417 kb) [file 10020_2019_104_MOESM2_ESM.pdf]

p.His267Tyr

```
Homo sapiens                  260 PAPPPPPHAYGYGPYGCGYGLQLPPYAPPSALFAAAAAAAAAAAFHPHSPP------PPP 313
Monodelphis domestica         289 PAPPPPPHAYGYGPYSCGYGLQLQPYPPPSALFAAFHPHPAAAHHHPHSHP-HSPPPPPP 347
Pan troglodytes               241 PAPPPPPHAYGYGPYGCGYGLQLPPYAPPSALFAAAAAAAAAAAFHPHSPPP-------P 293
Sus scrofa                    262 PAPPPPPHAYGYGPYGCGYGLQLPPYPPPSALFAAAA--AAAAAFHPHSPP------PPP 313
Cebus capucinus imitator      263 PAPPPPPHAYGYGPYGCGYGLQLPPYAPPSALFAAAAAAAAAAAFHPHSPP------PPP 316
Odobenus rosmarus divergens   249 PAPPPPPHAYGYGPYGCGYGLQLPPYAPPSALFAAAAAAAAAAAFHPHSPPPRRPPPPPP 308
Delphinapterus leucas         269 PAPPPPPHAYGYGPYGCGYGLQLPSYAPPSALFAAAAA--AAAAFHPHSPPP---PPPPP 323
```
